# Pain and Inflammation of Dental Origin

**Published:** 1892-06

**Authors:** 


### Pain and Inflammation of Dental Origin.

Dr. Hugenschmidt (*La Semaine Medicale*) admirably sums up the differential symptomatology and treatment of the two principal classes of pains of dental origin. In the first class, the cause of the pain is acute inflammation of the pulp of the tooth; in the second class, the cause of the pain is an inflammation of the periosteum covering the alveolus. The characteristic symptoms of acute pulpitis are, intense neuralgic pain in the region supplied by the fifth nerve, the point of the maximum intensity being at the root of the affected tooth which is the seat of the disease. The pain is increased by the inhalation of cold air, or taking into the mouth any hot or cold liquid, or bringing in contact with the tooth any hard substance, as the seeds of fruits.

The pain may be constant or intermittent; it is often lancinating in character, and frequently there will be found sensitive points on the temples, or just below the orbit of the affected side. Examination of the affected tooth will usually disclose a carious cavity which has extended so deep as to have reached the pulp cavity. The proper treatment is to wash out the cavity carefully by means of a stream of warm water injected from a small syringe, using also, if necessary, a stilette with a little bit of cotton wrapped about the end of it. After the cavity has been thoroughly emptied, a bit of cotton should be placed in the cavity after having been saturated with either of the following solutions: Menthol, 18 grs.; chloroform, 30 grs.; or, hydrochlorate of cocaine and hydrochlorate of morphia, each 4 grs., and creosote sufficient to make a paste of the consistency of cream.

If neither of the above mixtures can be obtained, pure creosote or carbolic acid may be employed. Great care must be taken to avoid burning the surrounding parts. A minute bit of cotton moistened with acid should be placed in the bottom of the cavity, and then the cavity should be filled with dry cotton; the last bit of cotton may be advantageously moistened with collodion, which will exclude the fluids in the mouth. When the pain is due to periostitis, it is different in character; the pains, though lancinating, are continuous, never intermittent, only ceasing when the inflammatory products have found their way to the surface.

The seat of the cause of the inflammation is usually a dead pulp. The tooth which is the seat of the inflammation becomes more and more sensitive, until it cannot be touched without severe pain, and the patient cannot eat on the affected side. The tooth is not sensitive to cold air, and very little or not at all sensitive to hot or cold liquids. During the first twenty-four hours the inflammation may sometimes be relieved by freely bathing the gum in the neighborhood of the affected tooth with equal parts of laudanum and tincture of iodine. Care must be taken not to apply the remedy too freely. Bathing the gum with a mixture consisting of 5 grams tincture of iodine, 1 gram tincture of aconite, 9 grains of hydrochlorate of cocaine is still more powerful in relieving the inflammation, but in the majority of cases the only means of radical cure will be removal of the pulp of the tooth or extraction of the tooth.

The important point is to recognize the difference between inflammation of the alveolar periosteum and an acute pulpitis.

